# “Now It's Just Old Habits and Misery”–Understanding the Impact of the Covid-19 Pandemic on People With Current or Life-Time Eating Disorders: A Qualitative Study

**DOI:** 10.3389/fpsyt.2020.589225

**Published:** 2020-10-27

**Authors:** Catherine McCombie, Amelia Austin, Bethan Dalton, Vanessa Lawrence, Ulrike Schmidt

**Affiliations:** ^1^Department of Health Services and Population Research, King's College London, Institute of Psychiatry, Psychology & Neuroscience, London, United Kingdom; ^2^Section of Eating Disorders, Department of Psychological Medicine, King's College London, Institute of Psychiatry, Psychology & Neuroscience, London, United Kingdom; ^3^South London and Maudsley NHS Foundation Trust, London, United Kingdom

**Keywords:** eating disorders, COVID-19, coronavirus, recovery, anorexia nervosa, social isolation

## Abstract

**Background:** Many aspects of the Covid-19 pandemic may make living with or recovery from an eating disorder (ED) particularly challenging. Understanding the processes which underlie the psychological and behavioral responses of people with EDs during this time are key to ensure tailored support in these unprecedented circumstances.

**Methods:** People with lifetime EDs (*n* = 32) were recruited through social media from May to June 2020 during a period of strict infection control measures in the United Kingdom (i.e., “lockdown,” “social distancing”). They completed open-ended questions in an online anonymous questionnaire that invited them to reflect on how various aspects of their lives have been affected by the Covid-19 pandemic, including ED symptoms and coping strategies. Responses were analyzed using thematic analysis.

**Results:** Most respondents reported that their ED worsened or resurfaced. Isolation, low mood, anxiety, lack of structure, disruption to routines, and media/social media messages around weight and exercise seemed to contribute to this. There was a clear sense that individuals struggled with which aspects of psychological distress to prioritize, i.e., mood vs. ED cognitions and behaviors, particularly as attempts to cope with one often exacerbated the other. Nonetheless, some participants reported “silver linings” of the pandemic.

**Conclusions:** In this self-selected sample, deterioration or recurrence of ED symptoms were the norm. This has implications for the provision of treatment and care for people with EDs both in the immediate short-term and in potential future waves of the pandemic, with a significant surge of new and re-referrals expected.

## Introduction

The coronavirus (Covid-19) pandemic has had a universal impact on mental health (1, 2), with a general increase in psychological distress, anxiety, and poor sleep (3). For those with pre-existing mental health conditions, such as eating disorders (EDs), the effects may be particularly profound (4). Responses to the pandemic at both the governmental and public level may make living with or recovery from an ED particularly challenging, and there has been much discussion about potential impacts on those with EDs (5–9). Hypothesized risk factors specific to these individuals include social isolation, changes in access to food, media messages around weight, limits on exercise, and reduced access to healthcare (10). High levels of worry, rumination and difficulties tolerating uncertainty, previously reported in those with EDs [e.g., (11–13)], may also play a role at a time when general population anxiety is higher (14).

Initial evidence suggests the Covid-19 pandemic is exacerbating symptoms for 15–69% of those with EDs (15, 16). A study in recently treated patients with severe anorexia nervosa reported reduced access to services, change in community activities, and heightened ED symptoms as key factors underlying this deterioration (17). However, an in-depth understanding of the experiences and perspectives, including the perceived mechanisms underlying any symptom changes, of people with EDs in the community across diagnoses and at different stages of recovery is needed. As we move toward easing of restrictions, it will be important to fully understand how people have been affected, what this means for life after lockdown, and how to better prepare to mitigate the impact of potential future pandemic control measures. Therefore, using an online survey, this study investigated the unique experiences and perceptions of people with past or present EDs living in the UK during the Covid-19 pandemic and associated lockdown restrictions.

## Materials and Methods

Ethical approval was granted by the King's College London Psychiatry, Nursing and Midwifery Research Ethics Subcommittees (Reference: LRS-19/20-18225).

### Participants

Adults (≥17 years) based in the UK and with a current or previous ED were eligible to take part. Participants were invited via posts on Twitter that contained a hyperlink to the anonymous survey. Responses were collected between 7th May and 12th June 2020. In total, 77 people accessed the survey. Of these, 75 were eligible, 53 of whom provided informed consent, and 49 provided data. Thirty-two participants submitted their responses to qualitative questions and were included in analyses (see Table 1 for participant demographics). In the whole sample (*n* = 32), 14 participants identified as having a current ED, 16 identified as in recovery, and two identified as recovered. Given the small sample of fully recovered participants, those in recovery and those recovered were grouped together for subgroup analyses.

**Table 1:** Participant demographics and clinical characteristics reported for the whole sample and also subdivided into those with a current eating disorder and those who identified as being in recovery/recovered.

	**Whole sample (*n* = 32)**	**Current ED (*n* = 14)**	**In recovery/recovered (*n* = 18)**
Age (years) (*M* ±*SD*)	35.2 ± 10.3	36.5 ± 10.8	34.1 ± 10.2
Diagnosis (*n*, %)			
AN	23 (71.9)	9 (64.3)	13 (72.2)
BN	3 (9.4)	2 (14.3)	2 (11.1)
BED	1 (3.1)	1 (7.1)	-
Other	5 (15.6)	2 (14.3)	3 (16.7)
Duration of illness (years) *(M* ±*SD*)	15.3 ± 10.3	16.0 ± 12.5	14.7 ± 8.2
Gender (*n*, %)			
Female	30 (93.6)	13 (92.9)	17 (94.4)
Male	1 (3.1)	1 (7.1)	-
Prefer not to say	1 (3.1)	-	1 (5.6)
Ethnicity (*n*, %)			
White	32 (100)	14 (100)	18 (100)
Living arrangement *(n*, %)			
Alone	9 (28.1)	2 (14.3)	7 (38.9)
With others	23 (71.9)	12 (85.7)	11 (61.1)
Country (*n*, %)			
England	27 (84.4)	10 (71.4)	17 (94.4)
Wales	1 (3.1)	1 (7.1)	-
Scotland	3 (9.4)	2 (14.3)	1 (5.6)
Northern Ireland	1 (3.1)	1 (7.1)	-
EDE-Q Global score (*M* ±*SD*)	3.3 ± 1.5	4.2 ± 1.2	2.7 ± 1.3
DASS-21 Total score (*M* ±*SD*)	32.4 ± 16.8	41.6 ± 14.5	25.2 ± 15.1

*ED, eating disorder; M, mean; SD, standard deviation; n, number; AN, anorexia nervosa; BN, bulimia nervosa; BED, binge eating disorder; EDE-Q, Eating Disorder Examination Questionnaire; DASS-21, Depression Anxiety and Stress Scales—Version 21*.

### Measures and Procedure

All participants provided informed consent before proceeding to the survey questions. They were then asked to provide demographic information, confirm their ED status (i.e., current/partial recovery/full recovery), and report relevant diagnoses and illness durations. Participants then completed the Eating Disorder Examination Questionnaire Version 6.0 [EDE-Q; (17)], a 28-item questionnaire assessing ED symptoms over the past 28 days. The EDE-Q contains four subscales (dietary restraint, eating concern, shape concern, weight concern) which combine into a global severity score (18). Participants also completed the Depression, Anxiety and Stress Scales–Version 21 [DASS-21; (19)], a 21-item questionnaire used to measure general psychopathology over the previous week. Each item is rated on a four-point scale from “did not apply to me,” to “applied to me very much, or most of the time.”

**Figure 1:**
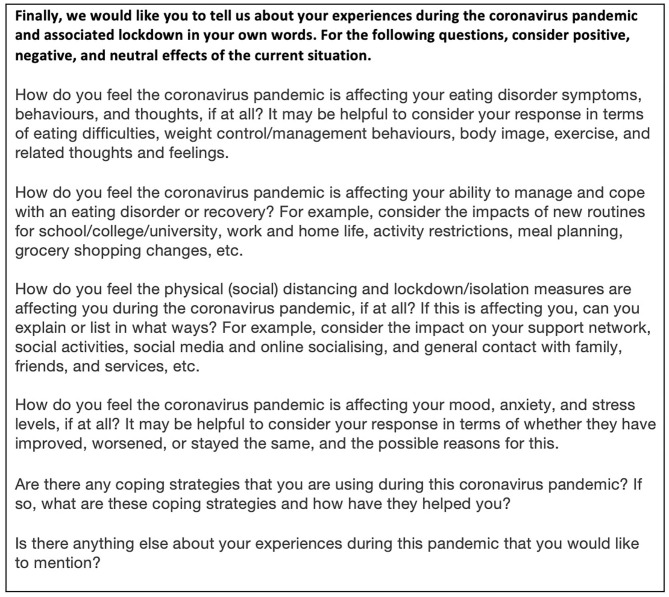
Open ended questions included in the survey.

Finally, six open-ended questions asked individuals to describe their experience of the pandemic in their own words (Figure 1). Questions covered disordered eating behaviors and thoughts; their ability to cope with and manage ED symptoms/recovery; effects of lockdown measures; effects on mood, anxiety and stress levels; and coping strategies used during the pandemic. There was also space to report anything else that they would like to mention. All questions, except those checking eligibility criteria, were optional.

### Data Analysis

Questionnaire responses were analyzed using thematic analysis, following the six phases described by Braun and Clark (20), taking an inductive approach. Analysis focused on understanding how aspects of the experience of the pandemic impacted upon people with past or present EDs to help inform support during and after the pandemic. Initial coding was conducted by one researcher (CM), with 30% of responses also coded by a second researcher (AA). Codes were developed through a process of line by line analysis, looking for concepts in the data related to the study aims and employing constant comparison across the dataset to see if these concepts recurred and/or formed patterns. A coding framework was developed on the basis of the first 15 responses and then applied to each and every response. Discussion of the coding framework and thematic development took place with three researchers reviewing the data (CM, AA, and BD), before discussion of the findings with the wider research team. Comparisons were made across recovery status (current or recovered/in recovery), but small diagnosis subgroups, other than for anorexia nervosa, prevented diagnosis subgroup comparisons.

## Results

Two overarching themes were generated from the analysis—mechanisms contributing to ED exacerbation, and positive aspects of lockdown. Where there are clear differences in the experiences of current vs. in recovery/recovered participants, these are highlighted. Otherwise, the themes reflect experiences across diagnosis and recovery status. Following the title of each theme/subtheme and in Table 2, the percentage of participants (within the whole sample) who referenced that theme is given, as an indicator of the pertinence of the theme within this sample.

**Table 2:** Percentages of participants who referenced each theme.

**Overarching themes**		**Sub themes**	
Mechanisms contributing to ED exacerbation	88%	Isolation	66%
		Worry, rumination, and worsening anxiety and depression	81%
		Media impact	47%
		Structure and routine	69%
Positive aspects of life in lockdown	72%		

### Mechanisms Contributing to ED Exacerbation (88%)

Distinct factors were identified by participants as directly contributing to deterioration in ED symptoms or relapse as a result of pandemic restrictions and associated changes in lifestyle. This theme consists of a number of subthemes, described below, that each contribute to the exacerbation of ED symptoms. Underlying this theme was the concept of balance and conflict: a constant struggle to balance coping with an ED or recovery while trying to effectively manage other aspects of life and mental health.

#### Isolation (66%)

The sense of physical and psychological isolation caused by lockdown measures affected ED symptoms in many ways. The lack of physical contact with and reassurance from others led to an increased sense of unease and difficulties in coping with ED cognitions–“*Video calls are not the same as a hug, or the reassurance of knowing my partner finds me attractive in my existing body, so it is easier to give in to the voices that say I need to drastically change”* (P12), “*I can't have a hug or hear and see them tell me I'm ok and safe when the eating disorder feels like it is winning”* (P1). Respondents reported that they had fewer distractions when staying at home (government-enforced lockdown) compared to in normal life, which led to an increase in rumination, ED thoughts and body-checking–“*More time alone to think about feelings/weight/food as unable to have usual social contact”* (P5)*, “I definitely walk past the mirror more often and do some body checking”* (P2).

Many respondents reported that not seeing loved ones, or healthcare services in person made it easier to hide worsening ED symptoms and weight changes–“*It can be very easy to hide weight loss and how you are really feeling when not seen in person”* (P1). Participants also reported significant challenges and barriers associated with communicating solely through remote/virtual methods, such as finding it difficult to be honest through video or phone calls, feeling disengaged and more distant, and more prone to lie about their weight to their clinical team–“*My weight is now dropping and I have been told if it continues to drop I will have to go into hospital which I don't want so I am now basically giving a false weekly weight over the phone”* (P4). Although support was technically there, many responses suggested an awareness that isolation was increased by virtual communication–“*I find video calls can be quite draining and I find it hard to be honest about how I am feeling on them”* (P11).

There was a strong sense among participants that the ED voice (an internal critical voice that commentates on shape, weight, and eating) is in the background even in those long recovered, and that coping with an ED and/or maintaining recovery represent a continual effort to suppress this voice. Lack of distractions, competing voices, and in-person support were key contributing factors in making the ED voice more prominent and harder to resist: “*It's not like it ever really goes away but when I'm busy I'm able to distract from it more”* (P18)*, “Not being able see family and friends is extremely difficult and makes it harder to tackle the ‘voice’ of anorexia when it appears”* (P13).

Balance between social activity and time for oneself was highlighted as a key issue, as most participants were living with others during the UK lockdown. For those who experienced changes in living situation due to the pandemic (e.g., previously living alone or with a different household), this was particularly challenging. Participants reported conflict around the need for space and privacy with the need for social support and contact. “*I'm feeling very bored and isolated - I'm normally very active but those things have shut down, and I dislike socializing online. I'm staying with family but finding myself becoming irritable and wanting space”* (P8).

The impact of isolation was referenced by many respondents as being difficult, and participants acknowledged that support from others, even across virtual platforms, has been essential in helping them cope. “*Reaching out to friends has helped a lot as I know others are struggling too and they can suggest things they are using to help”* (P14). Some also reported that trying to support others gave them a greater sense of purpose during lockdown–“*I also volunteer for a mental health charity so this has been good for my morale knowing I am helping in a small way”* (P22).

#### Worry, Rumination, and Worsening Anxiety and Depression (81%)

Participant responses shed light on how the interaction of depression and anxiety with ED symptoms had led to worsening overall psychological well-being during lockdown. Many reported that lack of enjoyable activities and social contact directly caused depressive symptoms–“*Without structure and focus (and meaning) of uni and friends and having a life that feels worth living, now it's just old habits and misery”* (P23). Several participants reported a subsequent increase in suicidal thoughts–“*Basically every day I wake up and I don't want to be here. I want to kill myself as most of the time I feel like there's no hope,” (P4)*.

For some, reduced stressors from normal life provided a relief from some anxiety, but the corresponding increase in mental space (i.e., time to think) worsened ED thoughts—“*The absence of usual stressors such as work has helped in a way but it's also caused eating disorder to increase in volume so it's a tricky one”* (P18). However, many people reported increases in generalized anxiety related to the global situation, including worries about work, family, friends, were reported to make tolerating anxiety around food harder, particularly in combination with having more time to ruminate than usual–“*The increase in general anxiety through the changes imposed on us has had the effect of raising the ‘volume’ of the voice inside me saying you can and can't have this or that to eat”* (P4). Generalized anxiety was one experience that seemed to differ between people in recovery/recovered and those with current EDs: higher levels of general anxieties were reported by the former group, particularly focused around work, family, and the general pandemic situation. Anxiety in the subgroup with a current ED was also high, but the content of reported worries tended to be more specifically associated with EDs.

A number of respondents also referenced anxiety around lockdown ending, with fear around a return to “normal” life and exposure to others' comments around their weight. For some, this was a source of internal conflict–“*I dread seeing people again and hearing them say that I've lost weight as a compliment and the horrible reinforcement of this battle in my mind, but I also fear that they won't say it”* (P19). Several participants referenced fear of their new-found balance being disrupted again as lockdown restrictions eased, with anxiety growing at the prospect of more changes–“*When lockdown was more stringent my anxiety was better, but now it has relaxed a bit my anxiety has returned”* (P18).

Participants reflected that coping strategies that helped with depression and anxiety directly exacerbated ED symptoms. Several reported that exercise helped depression, but made ED symptoms worse–“*If I don't run I struggle to manage my mood, but if I do run it makes managing the eating symptoms harder”* (P8). Worsening depression lead to one participant being prescribed anti-depressants, which they were worried would lead to weight gain and were reluctant to take, highlighting the conflict associated with managing different aspects of mental health.

#### Media Impact (47%)

The negative impact of both social media and news coverage was referenced by many participants. Focus around lockdown weight gain/loss on social media was identified as something making it harder to ignore ED thoughts–“*There's a lot of diet talk and staying active in lockdown content all over social media. It's hard to ignore and not feel influenced”* (P28). UK Government advice on exercise made participants feel both pressured to exercise and guilty for not using their allowed exercise time, even when they knew this would be unhelpful for them–“*Started exercise as the Government advice kept talking about the importance of getting exercise even though I'm underweight. I was trying to be healthy and use it to help gain weight i.e., strength training… but now I feel I have to do it every day and am not taking rest days” (P29)*. Media focus around exercise and compensating for food drove participants toward compulsive exercise–“*Without the constant daily exercise reminder I would never normally do any additional activity on days that I weight train”* (P28)–and strengthened ED-related thoughts—“*The news can make it seem dangerous to just eat, and I had only recently got to that point in recovery”* (P10).

#### Structure and Routine (69%)

Loss of control over day-to-day life caused by pandemic restrictions, contrasting with a need to maintain some sense of routine, impacted ED symptoms–“*I'm trying really hard not to set myself arbitrary rules around food to feel in control of SOMETHING during the pandemic”* (P19), “*I haven't had the external pressure to be flexible in my routine and therefore my restriction has become more rigid*” (P3). Increased free time presented another challenge in keeping ED behaviors at a healthy level for some, but for others this time allowed them to better focus on recovery-focused strategies “*I'm at home all the time so it's very easy to maintain a routine with meals*” (P8).

Disrupted routines meant finding a new balance, which proved challenging for many–“*It's made me realize that my structure and routine was keeping me safe and on track with recovery and now I've been forced to change my routine this has affected how I am managing recovery”* (P14).

### Positive Aspects of Life in Lockdown (72%)

This theme reflects participants' resilience in finding good in difficult times, and highlights how some aspects of the pandemic have been helpful in managing a past or present ED.

While lockdown was experienced as negative by the majority of participants, many still found aspects of it that enhanced their well-being and/or tried to focus on positive impacts of life in lockdown, even those struggling with current EDs. Participants reported having more space and time for healing and self-care, with less time taken up by commuting and normal day-to-day activities—“*I'm furloughed so have had the mental space to acknowledge that I do actually have a problem. I have started recognizing my own ED thought patterns and behaviors and am working on challenging them”* (P28).

Having less pressure to engage in social activities and fewer worries around things such as shopping, events, meetings, and work, was experienced as positive by many, and lead to a sense of ease and comfort in time out to spend at home–“*I have framed this time as an opportunity to catch up on reading and movies I have missed. With this in mind, it is very easy not to be negatively affected”* (P22). Some reported that physical distance from friends, family, and colleagues had improved relationships, made them reach out more to others for support, and become more involved in their community–“*I can, and do, bring food to elderly friends and can generally be one of the people who are looking after others. So I guess in that sense the pandemic has some unexpectedly empowering aspects”* (P10). Several participants identified relief at the halting of normal life due to lockdown, particularly of social activities—“*I feel a reduction in guilt for being an introverted loner, it's ok to be antisocial now”* (P7).

For three (out of four) participants who reported an autism diagnosis, several aspects of lockdown were experienced as positive–“*Loving lockdown in many respects. Less people around and less cars and planes”* (P5), “*work from home finally is actually a big improvement to my everyday well-being and work stress*” (P19). The world seeming more peaceful and simple was also appreciated by many in the broader sample. Others referenced their life had not changed much through the pandemic, highlighting that not all experienced significant negative changes–“*I feel that one reason I'm not especially affected by lockdown is that the world has come to meet me in living a fairly minimalistic life. It's perhaps less of a change for me than for many others”* (P10).

## Discussion

**Figure 2:**
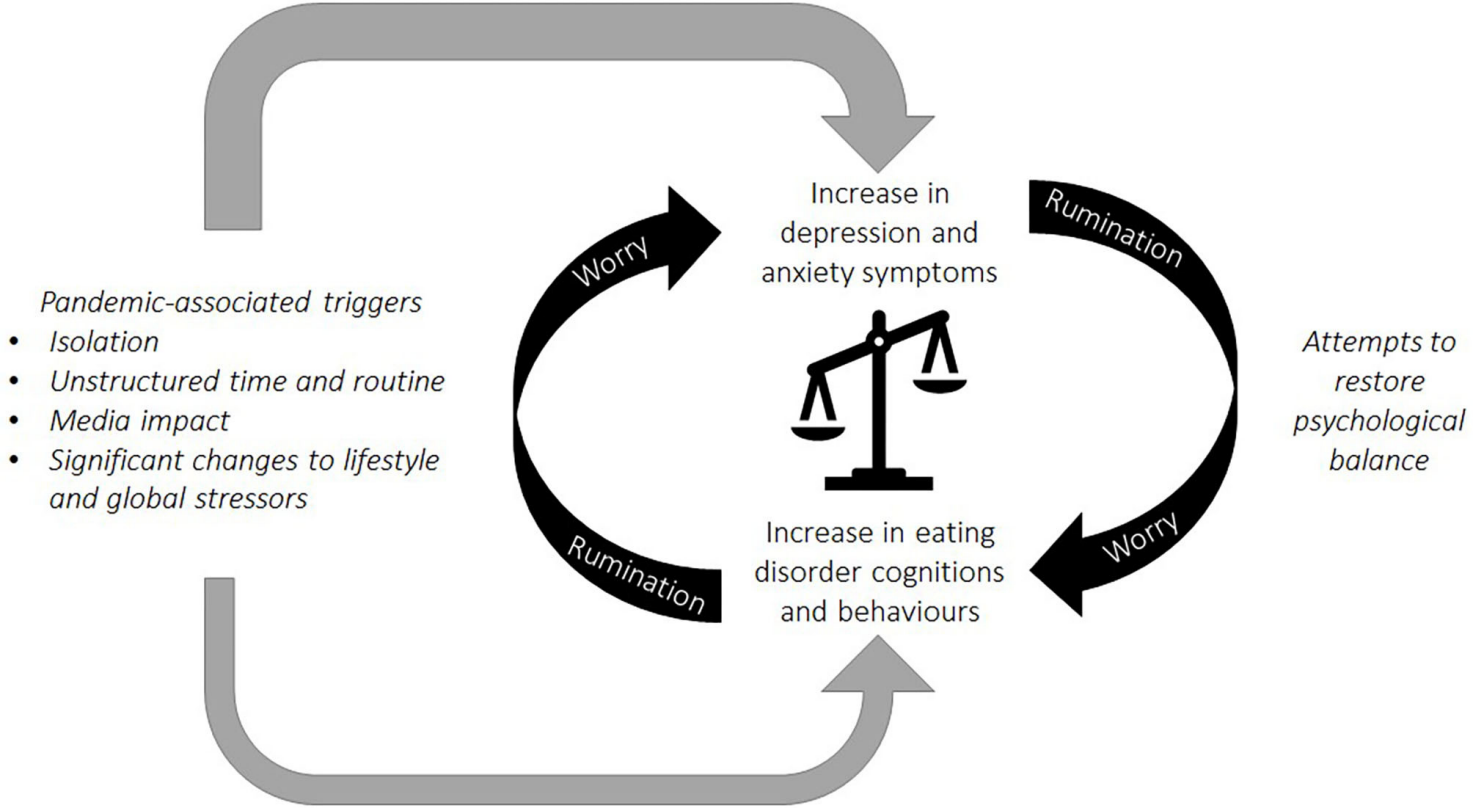
A conceptual model based on the reported interplay of stressors and outcomes. Experiences during the pandemic, including isolation, disruption to routine, and media messages were associated with a worsening of ED cognitions and behaviors, alongside an increase in symptoms of depression and anxiety. Symptoms of general and ED-specific psychopathology interacted in a vicious cycle and worry and rumination served to maintain this. Coping strategies used in an attempt to manage depression and anxiety often conflicted with, and exacerbated, ED symptoms, and vice versa.

Isolation, absence of valued elements of life, disruption to routine, and media triggers around the Covid-19 pandemic were seen as primary drivers of psychological distress in people with current and past EDs. Worries, rumination, and worsening anxiety and depression were identified as outcomes of Covid-19-related lifestyle changes, but also served as mechanisms contributing to ED deterioration in their own right. ED symptoms and depression and anxiety then appeared to interact in a vicious cycle, with worry and rumination seeming to play a key role in maintaining this. Attempts to manage depression and anxiety symptoms often conflicted with, and exacerbated, ED symptoms, and vice versa. Participants frequently reported that finding a balance between coping mechanisms was difficult to manage, which lead to increased feelings of guilt, shame, and depression. The observed interactions between factors impacting on distress, depression and anxiety, and ED symptoms are illustrated in Figure 2. Our findings add context to themes recently identified by individuals with EDs during the pandemic [e.g., (16, 21)], and highlight the universality of challenges experienced across ED diagnoses and stages of recovery during the pandemic. The findings also illustrate the additional burden of having pre-existing mental health conditions during a time of increased anxiety and depression among the general population (22, 23).

Difficulties around balancing conflicting priorities were experienced in a variety of ways by all participants, highlighting the psychological effort involved in managing an ED or maintaining recovery. Profound changes to daily life caused by the Covid-19 pandemic may have unbalanced psychological homeostasis in many people, resulting in attempts to restore equilibrium and reduce feelings of unrest (24). The findings support this idea, as participants seem to be striving toward psychological balance through the use of familiar adaptive and maladaptive (i.e., eating pathology, rumination) coping mechanisms, and through forming new routines to compensate for disruption of long-established routines. Challenges with this were experienced to a similar extent in both those recovered from or with a current ED. A prominent conflict described by respondents was of balancing the need for temporary relief from depression and anxiety, through behaviors such as binge eating and excessive exercise, with awareness of the negative impact of these on EDs. A harm reductionist approach, i.e., implementing practical strategies to reduce the damage to physical and mental health, as opposed to the traditional approach of promoting abstinence from ED behaviors, may be appropriate for some individuals during Covid-19 (25).

Isolation and unstructured time affected participants in many ways, and resulted in the ED voice becoming more prominent and harder to resist. Experience of the ED voice has been shown to influence ED pathology [e.g., (26)], such that a stronger ED voice has been associated with more severe ED psychopathology (27). This was reflected in the worsening of ED symptoms reported by the majority of participants, which is consistent with research showing that social support is an important factor in managing ED recovery (28, 29). Alongside physical isolation and increased volume of the ED “voice,” difficulties around virtual communication (e.g., feeling distant, easier to be disengaged) lead to participants feeling that they lacked meaningful support. This made it easier for participants to hide worsening ED symptoms and weight changes from loved ones and clinical teams. Given the accelerated the drive toward virtual treatment and support, this may have important implications for online treatment (9).

Both people recovered from and with current EDs reported positive experiences, or “silver linings”, associated with pandemic control measures, including more time for self-care and reduced social pressure. This active attempt to regulate emotion by cognitively reappraising an adverse experience (i.e., find “silver linings”) is not typically associated with EDs (30). High rates reported here, therefore, speak to the active effort of participants to cope. However, given the challenges faced in this pandemic, these attempts do not appear to have had significant impacts on overall well-being. The current study suggests an even greater need for support and services for those with EDs, as they appear to still be experiencing a high level of symptomatology despite active attempts to combat this. The impact of the pandemic among those with EDs will extend beyond dates of lockdown restrictions, and the findings of this study highlight some areas for future investigation and support.

## Strengths and Limitations

A key strength of this study is that it illustrates the difficulties experienced during the pandemic that appear applicable across ED type and recovery status. Asking detailed questions about experiences and enabling participants to respond in writing in their own time has provided rich and thoughtful responses, where participants have taken care to illustrate the many conflicting facets of their experiences. However, the sample is not representative of ethnic and gender diversity, and the recruitment strategy may have excluded people with limited access to internet, or who do not use social media. The small sample prevented subgroup analysis across diagnoses, which may have provided further insight into the commonalities and differences of experience across ED types. Additionally, findings cannot necessarily be generalized to other countries, as research by Termorshuizen et al. (16) has shown that experiences differ between countries, which may be an effect of differences in countries' responses to the pandemic. While the study only captures experiences at one point in time, thereby limiting understanding of how experiences may have changed over time, it provides a valuable basis for future investigations into the impact of the pandemic on people with past or present EDs.

## Conclusions

In this self-selected sample, deterioration or recurrence of ED symptoms were the norm. By identifying the key challenges experienced by people with past or present EDs during the lockdown phases of the pandemic, we have been able to highlight which aspects of this have been most impactful, and how this has contributed to ED symptom exacerbation and relapse. This has implications for the provision of treatment and care for people with EDs currently and also in future waves of the pandemic. ED services need to prepare for a significant surge of new and re-referrals, and to support patients with balancing the many aspects of mental health that have been negatively affected by the Covid-19 pandemic.

## Data Availability Statement

The raw data supporting the conclusions of this article will be made available by the authors, without undue reservation.

## Ethics Statement

The studies involving human participants were reviewed and approved by King's College London Psychiatry, Nursing and Midwifery Research Ethics Subcommittees. Written informed consent from the participants' legal guardian/next of kin was not required to participate in this study in accordance with the national legislation and the institutional requirements.

## Author Contributions

CM, AA, BD, VL, and US contributed to conception and design of the study. CM, AA, and BD performed the data analysis. CM and AA wrote the first draft of the manuscript. All authors contributed to the article and approved the submitted version.

## Conflict of Interest

The authors declare that the research was conducted in the absence of any commercial or financial relationships that could be construed as a potential conflict of interest.
